# Visualizing Glioma Infiltration by the Combination of Multimodality Imaging and Artificial Intelligence, a Systematic Review of the Literature

**DOI:** 10.3390/diagnostics11040592

**Published:** 2021-03-25

**Authors:** Sabrina Honoré d’Este, Michael Bachmann Nielsen, Adam Espe Hansen

**Affiliations:** 1 Department of Diagnostic Radiology, Copenhagen University Hospital—Rigshospitalet, 2100 Copenhagen, Denmark; Mbn@dadnet.dk (M.B.N.); Adam.espe.hansen@regionh.dk (A.E.H.); 2 Department of Clinical Medicine, University of Copenhagen, 2200 Copenhagen, Denmark

**Keywords:** artificial intelligence, glioma, glioblastoma, magnetic resonance imaging, multi-modality imaging, advanced imaging

## Abstract

The aim of this study was to systematically review the literature concerning the integration of multimodality imaging with artificial intelligence methods for visualization of tumor cell infiltration in glioma patients. The review was performed in accordance with the preferred reporting items for systematic reviews and meta-analysis (PRISMA) guidelines. The literature search was conducted in PubMed, Embase, The Cochrane Library and Web of Science and yielded 1304 results. 14 studies were included in the qualitative analysis. The reference standard for tumor infiltration was either histopathology or recurrence on image follow-up. Critical assessment was performed according to the Quality Assessment of Diagnostic Accuracy Studies (QUADAS2). All studies concluded their findings to be of significant value for future clinical practice. Diagnostic test accuracy reached an area under the curve of 0.74–0.91 reported in six studies. There was no consensus with regard to included image modalities, models or training and test strategies. The integration of artificial intelligence with multiparametric imaging shows promise for visualizing tumor cell infiltration in glioma patients. This approach can possibly optimize surgical resection margins and help provide personalized radiotherapy planning.

## 1. Introduction

Gliomas, especially high-grade gliomas (HGG), are one of the most frequent types of primary brain tumors with a median survival of only 15 months [1]. The current standard for diagnosis and prognosis relies on conventional Magnetic Resonance Imaging (MRI), especially T1 weighted contrast-enhanced (ce) and T2 weighted/FLAIR (Fluid attenuated inversion recovery) MRI [2,3]. Whilst contrast enhanced MRI can help delineate tumor margins it is not sufficient in depicting low-density tumor cell infiltration beyond the contrast enhancing region, which is commonly occurring for high-grade gliomas [4,5,6]. Infiltrating tumor cells, which may even be present in brain regions without any MRI abnormalities, could play an important role for the limited HGG treatment efficacy [7,8]. Indeed, eventually the vast majority of high-grade brain tumors will recur [9,10]. The shortcomings of conventional MRI with regard to the diffuse infiltrative growth pattern of HGG, along with the extensive intratumoral heterogeneity, makes tumor delineation and the following surgical resection and radiotherapy planning challenging [6,11]. 

Recently, advanced imaging techniques have been explored as a way to help better the detection and delineation of diffuse glioma infiltration in the non-enhancing tumor region [12,13,14,15,16,17]. This includes additional parameters available from perfusion-weighted MRI regarding image neovascularization as well as permeability and microscopic information on tissue architecture from diffusion tensor imaging (DTI) [18,19]. However, single imaging modalities have only achieved moderate accuracy for the detection of infiltration [20]. In acknowledgement of this, various groups have focused their attention on an integration of multiple imaging modalities in a predictive model showing the probability of tumor presence or later tumor recurrence in glioma patients [21,22,23,24,25,26,27,28,29,30,31,32]. By the aid of artificial intelligence (AI) methods a variety of imaging parameters can be considered simultaneously as opposed to a manual visual assessment of tumor extent during treatment planning. Such a combination of imaging modalities into a predictive model may aid delineation of tumor infiltration and provide a more relevant target for surgical resection and radiotherapy [33].

The aim of this systematic review was to provide an overview of the literature concerning the combination of multimodality imaging with AI to create a visualization of glioma tumor cell infiltration. We focused on the diagnostic accuracy for detecting infiltration through a predictive model, which MRI and supplementary modalities were combined and what methodologies including artificial intelligence methods were applied.

## 2. Methods

This systematic review was completed in accordance with the preferred reporting items for systematic reviews and meta-analysis (PRISMA) guidelines [34].

The literary search was conducted on the 28th of August 2020 in multiple databases; PubMed, Embase, The Cochrane Library and Web of Science. The following search string was used in all four databases:

(((((((((((((ai artificial intelligence) OR (machine learning)) OR (deep learning)) OR (support vector machines)) OR (radiomics)) OR (image interpretation)) OR (multiparametric)) OR (multimodal)) OR (image processing)) OR (algorithm))) AND (MRI)) AND (((glioma) OR (Glioblastoma)))) AND (((((infiltration) OR (invasion)) OR (recurrence)) OR (“biopsy”)) OR (“biopsies”)).

The search string was constructed with four components covering the computational, the MRI, the tumor type and the pathologic aspects. A large variety of keywords were found necessary for the computational search component, since a vast methodology is found within this field. Publications not written in English and/or published earlier than the 1st of January 2010 were excluded from the study.

Inclusion criteria were studies, that combined multiparametric imaging (defined as ≥2 types and must include MRI) to create a visualization of tumor infiltration in glioma patients exclusively. The visualization had to be presented as a map or likewise, showing the probability of tumor presence or later tumor recurrence. Furthermore, all studies must have a reference standard for tumor infiltration, which could be either histopathology from a stereotactic biopsy or tumor recurrence on follow up imaging. Studies focused on imaging of specific molecular markers such as but not limited to gene expressions, proteins or immunophenotyping, within neurooncology were not included. As well, exclusion criteria were publications concerning a single imaging modality, multiple imaging modalities which were not combined to predict infiltration and all pre-clinical studies.

**Figure 1:**
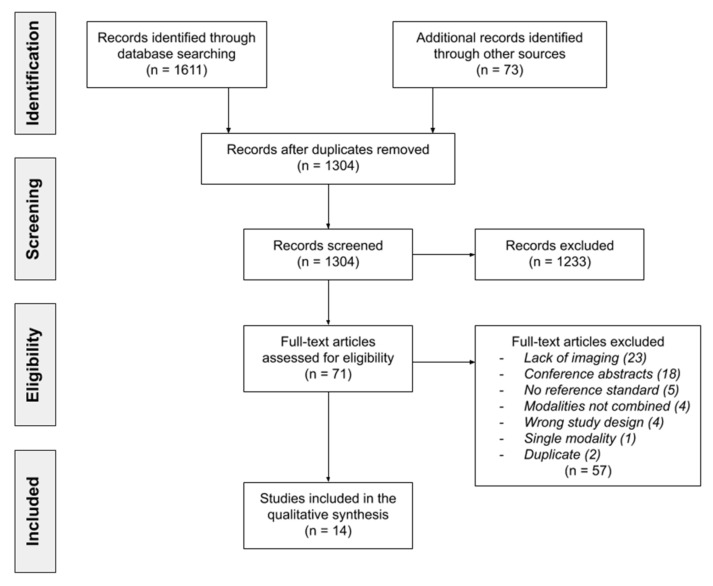
Preferred Reporting Items for Systematic Reviews and Meta-analysis (PRISMA) flowchart of the literature search and study selection.

All results from the search strategy were managed in the Cochrane technology platform, Covidence [35]. After the removal of duplicates the articles were screened by title and abstract by two authors (S.H.D. and A.E.H.). If the abstract was inconclusive about an article’s eligibility the study was included in the full text screening. Full text screening was performed by the before mentioned same two authors using the same eligibility criteria. In case of disagreement a consensus was reached. The literature search and study selection process are summarized in the PRISMA flowchart in Figure 1. The initial search provided 1231 publications, 1163 of which were excluded during title and abstract screening. The reference list of the remaining 68 publications were additionally screened by title and 73 articles, identified through these reference lists, were further screened by abstract using the eligibility criteria previously mentioned. A total of 71 studies were screened by full text and 14 articles were included in this review.

The extracted data from the included studies were number of participants, imaging modalities, tumor segmentation method, tumor classification, training and test strategy, artificial intelligence method, reference standard, pathologic marker and statistical evaluation of model performance. Imaging modalities which were obtained in the study but ultimately not retained in the predictive model, either for tumor segmentation or training of the model, were not presented amongst the results. Furthermore, the main table included only the acquired imaging modalities; any derived maps or data, such as relative cerebral blood volume (rCBV), apparent diffusion coefficient (ADC), fractional anisotropy (FA), etc., were not presented, but is available in Appendix A. The included studies did not provide comparable data; therefore, no meta-analysis was performed.

The quality and bias assessment of the included studies was performed by the two authors (S.H.D. and A.E.H). It was conducted using the Quality Assessment of Diagnostic Accuracy Studies (QUADAS2), which evaluate the studies in 4 domains (patient selection, index test, reference standard and flow and timing) regarding risk of bias and concerns for applicability [36]. Lowest risk of bias and concern for applicability is preferable. A consensus regarding the risk of bias and concerns for applicability was reached between the two authors on all studies.

A lack of specification of consecutive or other type of patient selection was considered as an unclear risk of bias. The reference standard for diagnostic accuracy studies involving AI prediction models, is generally employed both during model training and testing, with a potential risk of bias. Thus, the authors chose the following division regarding risk of bias of the index test: A lack of independent diagnostic accuracy testing of model performance resulted in a high risk of bias of the index test. Use of cross validation for this purpose was classified as an unclear risk of bias while having a separate validation cohort for diagnostic accuracy testing, was assessed as the optimal strategy and resulted in a low risk of bias. As well, manual delineation of tumor infiltration as a reference standard was categorized as a low risk of bias since this along with histopathological confirmation was accepted in the inclusion criteria. The applicability of the reference test was marked as unclear when RANO criteria for Gliomas (Response Assessment in Neuro-Oncology), which defines progression but not necessarily infiltration, was used as reference standard.

## 3. Results

### 3.1. Study Characteristics

14 studies were eligible for this review. Eight of these were retrospective studies [21,22,23,27,30,31,32,37] and six were prospective [24,25,26,28,29,38]. Two of the reported six prospective studies had clear specification of being consecutive [24,29]. The focus of all studies was high-grade glioma, with two studies including also low-grade glioma [24,29]. All studies except Durst et al. [24] incorporated two or more conventional MRI sequences (T1, T1ce, T2, FLAIR) in their final model. These were primarily used for tumor segmentation and image alignment. The most frequent integrated advanced modalities were diffusion tensor imaging (DTI) and dynamic susceptibility contrast (DSC), being used in the model of nine and seven studies, respectively, and appearing in combination in six studies [24,25,26,30,31,32]. Three studies incorporated FET-PET scans in their predictive model [27,28,29], one including also FDG-PET [28].

A stereotactic biopsy as reference standard was applied in six studies, five of which had an experienced neuropathologist assessing tumor presence through tumor nuclei count or cell density [24,25,26,29,38]. Chang et al. used cell density estimated by a fully automated cell-counting algorithm designed by the same group [37]. The remaining eight studies hence used imaging follow-up as a reference standard. One study relied on the RANO criteria for progression as a marker of pathology [22], while the remaining seven used manual delineation [21,23,27,28,30,31,32]. In four studies, the delineation was performed by ≥2 experts [28,30,31,32]. Anwar et al. had an experienced neuroradiologist perform the delineation in concurrence with a multi-interdisciplinary tumor board [21]. Blumenthal et al. and Chang et al. [22,23] relied on the delineation of a single radiologist while Lipkova et al. did not specify by who the delineation was performed [27].

Four of the included studies provided measures of diagnostic accuracy of model performance by testing the predictive models on an independent validation patient cohort [30,31,32,38]. Of the remaining ten studies, five used leave one out cross validation (LOOCV) and one study used two-fold cross validation (TFCV) to estimate the accuracy of model performance [21,22,25,26,28,29]. Three studies did not provide an independent accuracy test to assess model performance in practice but calculated a correlation between actual and predicted cell counts [24,37] or tested their proposed model within the primary training cohort [23]. Finally, Lipkova et al. performed a visual comparison between the outlines of the predicted and the delineated tumor recurrence, despite having an independent validation cohort [27].

Included studies with main results are listed in Table 1. Extracted supplementary data is listed in Appendix A.

### 3.2. Study Findings

The included studies all concluded their findings to be of significant value for future clinical practice. The proposed prediction models were evaluated based on their presented area under the curve (AUC) for visualization of tumor infiltration. An AUC was available in six of the included 14 studies, with AUC ranging from 0.74–0.91 [21,23,28,29,30,31]. There was no clear pattern between achieved AUC and included modalities. The eight studies with no available AUC provided alternative parameters for model performance. This included a sensitivity of 80.0%–100% and a specificity of 69.2%–100% in three studies [22,32,38] with two studies including also an accuracy of 78%–81.8% [32,38]. Four studies reported a Pearson’s correlation coefficient of 0.74–0.88 [24,25,26,37], while the final study provided no quantitative evaluation parameter of model performance [27].

### 3.3. Quality Assessment

The QUADAS2 consensus within each domain for studies performing diagnostic accuracy testing on a separate validation cohort, those performing cross-validation and those with no independent diagnostic accuracy test of model performance, are listed in Table 2, Table 3 and Table 4.

## 4. Discussion

This systematic review shows that the combination of multimodality imaging with artificial intelligence (AI) can visualize glioma infiltration. The predictive models were presented as maps, showing the probability of tumor presence or later recurrence in glioma patients. There was no consensus amongst the included studies regarding the best methodology to achieve this goal, although the inclusion of diffusion tensor imaging and dynamic susceptibility contrast MRI in addition to conventional MRI was predominantly used. The heterogeneity of incorporated modalities, reference standards and applied artificial intelligence method made a systematic comparison between studies challenging. Moreover, not all studies reported a diagnostic performance parameter of their findings making a quantitative evaluation difficult. However, moderate to high diagnostic accuracy was shown in many studies and all included studies concluded their findings to be of significant value for future clinical practice.

The detection and delineation of tumor infiltration beyond radiologically visible anatomic abnormalities, which are currently used for surgical resection and radiotherapy planning, could have profound implications for treatment management of glioma patients [39]. A visualization of tumor infiltration enables a tumor specific personalized surgical resection extending outside the contrast-enhancing region, or potentially even into normal appearing brain tissue. As well, an individualized risk-adapted radiotherapy plan, including a prediction of tumor presence or recurrence, could be envisaged to optimize local tumor control and minimize toxicity. However, further studies concerning the optimal methodology in visualizing tumor infiltration is still needed. While this study illustrates the capabilities of the combination of AI with multiparametric imaging for detection of tumor infiltration, the eventual impact on patient prognosis by implementing a predictive model during curative treatment planning would need to be addressed. Although promising, the included studies show no insight as to whether their results would lead to an improvement of the current survival of glioma patients. Therefor future randomized controlled trials, providing clarification of the potential treatment effect are desired. The field of medical imaging is constantly expanding and finding the optimal treatment strategy amongst existing and newly discovered methods, while balancing high diagnostic accuracy and cost–benefit could prove challenging.

Previous reviews and meta-analyses have focused on single modality imaging, conventional and/or advanced, in visualizing tumor cell infiltration in glioma patients [20,33,40]. The systematic review presented here extends the findings of earlier studies by revealing a diagnostic value in combining imaging modalities to detect tumor cell infiltration and predict tumor presence or recurrence (see Table 1). Moreover, amongst the included studies, several groups found added value compared to conventional imaging or any single modality [23,26,29,31,37,38].

The included studies employed a variety of AI algorithms to estimate predictive maps from the multiparametric imaging data. Algorithms encompassed computationally simple linear or logistic regression models [21,23,24,26,28,29,37], machine learning strategies as support vector machines [22,30,31], Bayesian modeling [27] and even convoluted neural networks [32]. Machine learning and neural network approaches can provide model flexibility with respect to complex multiparametric imaging data sets, possibly increasing the predictive performance. However, they are also more difficult to implement and may require a larger amount of training data, as compared to regression approaches. As well, incorporating biophysics of tumor infiltration as is seen with the mechanistic-proliferation model may aid model predictions even further [25].

Although all included studies found significant value to own results, only ten studies provided a separate validation cohort [30,31,32,38] or cross-validation [21,22,25,26,28,29] to calculate a prediction accuracy of the proposed model. The four remaining studies did not undertake any independent test of diagnostic accuracy, possibly reducing the significance of their findings [23,24,27,37].

The small sample sets available makes the development and testing of a predictive model within glioma patients challenging. All of the included studies had a limited amount of data (8–37 patients) to train their model upon thereby increasing the risk of overfitting of the AI algorithm [41,42]. As well, scans were incorporated from a single centre in all studies, thus restricting model training to the specific imaging machinery, quality and protocol at that given location. One study provided a separate validation cohort from a different hospital than the training cohort, thereby reducing the risk of overfitting [32]. A common database across locations, could provide a larger data set, wider range of modalities for model inclusion and a variation in image quality upon which to train a predictive algorithm and possibly creating a larger scale model better equipped for detecting the vast heterogeneity of high-grade glioma infiltration [43].

In this study we accepted manual delineation on follow-up recurrence scans as a valid reference standard for tumor infiltration. Although performed by radiological experts in seven of the eight included studies using follow-up imaging [21,22,23,28,30,31,32], the subjective element of manual delineation will always pose an inherent challenge [44]. As well, anatomical displacement between pre- and post-operative scans with regard to mass effect and edema can possibly complicate image registration in studies with predictions from pre-operative imaging [30,31,32]. A sub-optimal image registration of the predicted tumor presence or recurrence map and the follow-up images could challenge the model evaluation. Two groups reported this an issue and sought to provide the best approximate delineation of tumor infiltration on pre-operative scans, corresponding to the pathology proven recurrence on follow-up imaging, possibly increasing risk of bias [30,31]. The use of stereotactic biopsies provides a histopathologic confirmation of tumor presence thereby minimizing the subjective element of manual delineation as the reference standard. However, there will be ethic limitations regarding stereotactic biopsies beyond the enhancing and non-enhancing tumor region, complicating the visualization of tumor cell infiltration in radiologically normal appearing brain tissue. The manual delineation of recurrence on follow-up imaging provides an opportunity for easier applicability to whole brain predictions, not limited to radiologically visible abnormal anatomic regions. In this review studies predominantly restricted their visualization of tumor cell infiltration to the peritumoral and radiotherapeutic target regions [22,23,24,25,26,27,28,30,31,32,37,38], although two groups provided whole brain predictive maps [21,29].

Implementing a predictive model relying on advanced imaging techniques in daily practice comes with a challenge. Diagnosis and treatment monitoring of gliomas currently utilizes conventional MRI which is readily available and changing standardized routine clinical protocols to include advanced imaging and analysis will require an implementation effort. An example of such an effort is the existing software platform CaPTk (Cancer Imaging Phenomics Toolkit), that integrates advanced tools for analysis of radiographic cancer images, thereby alleviating the translation of artificial intelligence into medical image analysis in daily practice [45,46,47,48].

This systematic review focused on imaging of tumor cell infiltration, therefore studies providing no visualization of their predictive model were excluded. Likewise, studies focusing solely on visualization of tumor specific molecular markers, amongst these IDH (isocitrate dehydrogenase gene) mutation, EGFR (Epidermal Growth Factor) gene amplification and the proliferation biomarker Ki-67, were not included despite promising results in earlier studies [49,50,51]. Furthermore, studies aimed at predicting O6-Methylguanine-DNA methyltransferase (MGMT) promotor methylation status were not included, although showing promise as a prognostic biomarker for the alkylating chemotherapy treatment effect [52]. Another relevant field of interest concerns prediction mapping of various immunophenotypes within the tumor region which can possibly be used to provide a patient prognosis [53]. The newest World Health Organization (WHO) classification of gliomas incorporates molecular markers in the pathological diagnosis, making any of these markers relevant objects for future research concerning visualization of specific tumor subtypes [54].

## 5. Conclusions

In this review we demonstrated that integration of artificial intelligence with multiparametric imaging is a promising method for visualizing tumor cell infiltration in glioma patients. The proposed predictive models were presented as maps showing the probability of tumor presence or later recurrence both within the enhancing and non-enhancing regions and moderate to high diagnostic accuracy could be obtained. There is still a need for future studies concerning the best methodology in achieving the goal of visualizing tumor cell infiltration. Furthermore, larger cohorts in future prospective studies would increase diversity to the dataset and improve model performance. Although challenging, implementation of such a model can possibly optimize surgical resection margins and help provide personalized radiotherapy planning.

## Figures and Tables

**Table 1:** Results from all 14 included studies.

Author	Participants Training/Validation	Modalities	AI Method	Patholovgy	Reference Standard	Pathologic Marker	AUC Training/Validation	Conclusion
Akbari et al., 2016 [30]	31/34 *(Retrospective)*	T1, T1ce, T2, FLAIR, DTI, DSC	SVM	HGG (GBM)	Follow-up imaging	Manual delineation	0.80/0.84	Multiparametric MRI can elucidate patterns of tumor infiltration within peritumoral region that predict tumor recurrence
Hu et al., 2015 [38]	11/7 *(Prospective)*	T1ce, T2, DSC	DLDA, (DQDA, SVM)	HGG (GBM)	Biopsy	Tumor nuclei	NA	Multiparametric MRI and texture analysis can help characterize and visualize GBM’s spatial histologic heterogeneity
Rathore et al., 2018 [31]	31/59 *(Retrospective)*	T1, T1ce, T2, FLAIR, DTI, DSC	SVM	HGG (GBM)	Follow-up imaging	Manual delineation	0.83/0.91	Multiparametric MRI can assist in *in vivo* estimation of the spatial extent and pattern of tumor recurrence in peritumoral edema
Yan et al., 2020 [32]	37/20 *(Retrospective)*	T1, T2, FLAIR, DTI, DSC, Spectroscopy	CNN	HGG (GBM)	Follow-up imaging	Manual delineation	NA	Application of distinct imaging characteristics can potentially identify site of tumor progression.
Anwar et al., 2017 [21]	24 *(Retrospective)*	T1ce, FLAIR, DWI, DTI, Spectroscopy	Multinomial logistic regression	HGG (GBM)	Follow-up imaging	Manual delineation	0.75	Integrating advanced MRI with dosimetry can identify voxels at risk for progression
Blumenthal et al., 2017 [22]	32 *(Retrospective)*	T1, T1ce, FLAIR, DCE	SVM	HGG (GBM, astrocytoma, oligodendroglia)	Follow-up imaging	RANO	NA	Proposed Segmented RANO criteria classifies tumor and nontumor parts within enhancing and non-enhancing lesion.
Gaw et al., 2019 [25]	18 *(Prospective)*	T1ce, T2, DTI, DSC	SSL + mechanistic proliferation-invasion model	HGG (GBM)	Biopsy	Cell density	NA	Predictive model can provide patient-specific spatial maps of tumor cell density
Hu et al., 2019 [26]	18 *(Prospective)*	T1, T1ce, T2, DTI, DSC	Multivariable linear regression	HGG (GBM)	Biopsy	Tumor cell density	NA	Transfer learning optimizes tumor cell density models with particularly high predictive value in non-enhancing infiltrative tumor region
Lundemann et al., 2019 [28]	9 *(Prospective)*	T1, T1ce, T2, FLAIR, DTI, DCE, FET-/FDG-PET	Binomial logistic regression	HGG (GBM)	Follow-up imaging	Manual delineation	0.77	Model provides patient-specific maps of voxel-wise probability of recurrence.
Verburg et al., 2020 [29]	20 *(Prospective)*	T1, T1ce, T2, DTI, FET-PET	Generalized linear mixed model + Akaike	LGG, HGG	Biopsy	Neuropathologic assessment of presence of tumor	0.89 enhancing	Voxel-wise prediction model is more accurate to detect glioma infiltration than standard MRI in enhancing gliomas
Chang et al., 2017 [23]	26 *(Retrospective)*	T1, T1ce, FLAIR, DWI	Multivariable logistic regression	HGG (GBM)	Follow-up imaging	Automated segmentation with manually edited delineation	0.74	Likelihood of recurrence can be estimated as a function of voxel-wise signal intensity.
Chang et al., 2017 [37]	28 *(Retrospective)*	T1, T1ce, FLAIR, DWI	Multivariable linear regression	HGG	Biopsy	Cell density	NA	Correlation found between voxel-level signal intensity and cell density can provide mapping of intratumoral heterogeneity
Durst et al., 2014 [24]	10 *(Prospective)*	T1ce, DTI, DSC	Multivariate regression	LGG, HGG	Biopsy	Nuclear density	NA	Multiparametric voxel-based model may be able to more accurately predict infiltrative edge of tumor.
Lipkova et al., 2019 [27]	8 *(Retrospective)*	T1ce, FLAIR, FET-PET	Bayesian machine learning	HGG (GBM)	Follow-up imaging	Manual delineation	NA	Prediction of tumor cell density through multiparametric MRI and computational tumor growth model

AI: Artificial intelligence, AUC: Area under the curve, DTI: Diffusion Tensor Imaging, DSC: Dynamic Susceptibility Contrast, DCE: Dynamic Contrast Enhanced-MRI, DWI: Diffusion weighted imaging, FET-PET: 18F-fluoro-ethyl-tyrosine PET, FDG-PET: fluorodeoxyglucose-PET, SVM: Support vector machine, DLDA: Diagonal Linear Discriminate Analysis, DQDA: Diagonal Quadratic Discrimate Analysis, CNN: Convoluted neural network, SSL: Semi-Supervised Learning, HGG: High-Grade Glioma, GBM: Glioblastoma, LGG: Low-Grade Glioma, RANO: Response Assessment in Neuro-Oncology, NA: Not available.

**Table 2:** QUADAS2. Diagnostic accuracy test on separate validation cohort.

Study	Patient Selection		Index Test		Reference Standard		Flow and Timing
	*RoB*	*CrA*	*RoB*	*CrA*	*RoB*	*CrA*	*RoB*
Akbari et al., 2016 [30]	Unclear	Low	Low	Low	High	Low	Low
Hu et al., 2015 [38]	Unclear	Low	Low	Low	Low	Low	Low
Rathore et al., 2018 [31]	Unclear	Low	Low	Low	High	Low	Low
Yan et al., 2020 [32]	Unclear	Low	Low	Low	Low	Low	Low

RoB: Risk of Bias, CrA: Concerns for applicability.

**Table 3:** QUADAS2. Diagnostic accuracy test by cross-validation.

Study	Patient Selection		Index Test		Reference Standard		Flow and Timing
	*RoB*	*Cra*	*Rob*	*Cra*	*Rob*	*Cra*	*Rob*
Anwar et al., 2017 [21]	Unclear	Unclear	Unclear	Low	Low	Low	Low
Blumenthal et al., 2017 [22]	Unclear	Low	Unclear	Low	Low	Unclear	Low
Gaw et al., 2019 [25]	Unclear	Low	Unclear	Low	Low	Low	Low
Hu et al., 2019 [26]	Unclear	Low	Unclear	Low	Low	Low	Low
Lundemann et al., 2019 [28]	Unclear	Low	Unclear	Low	Low	Low	Low
Verburg et al., 2019 [29]	Low	Low	Unclear	Low	Low	Low	Low

RoB: Risk of Bias, CrA: Concerns for applicability.

**Table 4:** QUADAS2. No independent diagnostic accuracy test of model performance.

Study	Patient Selection		Index Test		Reference Standard		Flow and Timing
	*RoB*	*CrA*	*RoB*	*CrA*	*RoB*	*CrA*	*RoB*
Chang et al., 2017 [23]	Unclear	Low	High	Low	Low	Low	Low
Chang et al., 2017 [37]	Unclear	Low	High	Low	High	Low	Low
Durst et al., 2014 [24]	Low	Low	High	Low	Low	Low	Low
Lipkova et al., 2019 [27]	Unclear	Low	High	Low	Unclear	Low	Low

RoB: Risk of Bias, CrA: Concerns for applicability.

## Data Availability

No new data were created or analyzed in this study. Data sharing is not applicable to this article.

## Appendix A

**Table A1:** Supplementary extracted data, not crucial to the main table.

Author	Prediction Region	Derived Parameter Maps	Test of Model Performance	Sensitivity	Specificity	Accuracy	r
Akbari et al., 2016 [30]	Peritumoral edema	FA, RAD, AX, AT, rCBV	Independent validation cohort	91.18%	93.48%	91.25%	-
Hu et al., 2015 [38]	Peritumoral	rCBV	Independent validation cohort	100%	69.2%	81.8%	-
Rathore et al., 2018 [31]	Peritumoral edema	FA, RAD, AX, ADC, rCBV	Independent validation cohort	97.06%	76.73%	89.54%	-
Yan et al., 2020 [32]	Peritumoral edema	FA, ADC, rCBV, Cho/NAA	Independent validation cohort	80%	97.7%	78%	-
Anwar et al., 2017 [21]	Whole brain	FA, ADC, Cho/NAA	LOOCV	-	-	-	-
Blumenthal et al., 2017 [22]	Lesion area	*V_p_*, *K*^trans^, BAT	TFCV	100%	100%	-	-
Gaw et al., 2019 [25]	Peritumoral	MD, FA, rCBV	LOOCV	-	-	-	0.838
Hu et al., 2019 [26]	Peritumoral	MD, FA, rCBV	LOOCV	-	-	-	0.88
Lundemann et al., 2019 [28]	Radiotherapeutic region	MD, FA, F, V_b_, V_e_, K_i_, MTT	LOOCV	-	-	-	-
Verburg et al., 2020 [29]	Whole brain	ADC, FA	LOOCV	-	-	-	-
Chang et al., 2017 [23]	Peritumoral	ADC	Testing within primary training cohort	-	-	-	-
Chang et al., 2017 [37]	Peritumoral	ADC	Correlation	-	-	-	0.74
Durst et al., 2014 [24]	Peritumoral edema	MD, FA, *K*^trans^	Correlation	-	-	-	0.75
Lipkova et al., 2019 [27]	Peritumoral	-	Visual comparison on independent validation cohort	-	-	-	-

FA: Fractional anisotropy, RAD: Radial Diffusivity, AX: Axial diffusivity, AT: Axial trace, rCBV: relative Cerebral Blood Volume, ADC: Apparent Diffusion Coefficient, Cho/NNA: Cholin-to-NNA ratio/index, *V_p_*: Plasma volume, *K*^trans^: Volume transfer konstant, BAT: Bolus Arrival Time, MD: Mean diffusivity, F: Blood flow, V_b_: Intravascular blood volume, V_e_: Extra-vascular extra-cellular space volume, K_i_: Maps of vascular permeability, MTT: Mean Transit Time, LOOCV: Leave-one-out-cross-validation, TFCV: Two-fold-cross-validation, r: Pearson’s correlation coefficient.
